# Supplementary material for: OpenMM 7: Rapid development of high performance algorithms for molecular dynamics
Source: PLoS Comput Biol. 2017 Jul 26;13(7):e1005659. doi: 10.1371/journal.pcbi.1005659 (PMC5549999; doi:10.1371/journal.pcbi.1005659)
Supplement: S1 Source Code — (ZIP) [file pcbi.1005659.s002.zip › OpenMM-7.0.1-Source/docs/api-c++/generated/OpenMM.AmoebaTorsionTorsionForce.html]

AmoebaTorsionTorsionForce — OpenMM @OPENMM\_MAJOR\_VERSION@.@OPENMM\_MINOR\_VERSION@ documentation


# AmoebaTorsionTorsionForce¶

*class* `OpenMM::``AmoebaTorsionTorsionForce`¶
:   This class implements the Amoeba torsion-torsion interaction.

    To use it, create an `AmoebaTorsionTorsionForce` object then call `addTorsionTorsion()` once for each torsion-torsion. After a torsion-torsion has been added, you can modify its force field parameters by calling `setTorsionTorsionParameters()`.

    Methods

    |  |  |
    | --- | --- |
    | `AmoebaTorsionTorsionForce` | Create an `AmoebaTorsionTorsionForce`. |
    | `getNumTorsionTorsions` | Get the number of torsion-torsion terms in the potential function |
    | `getNumTorsionTorsionGrids` | Get the number of torsion-torsion grids |
    | `addTorsionTorsion` | Add a torsion-torsion term to the force field. |
    | `getTorsionTorsionParameters` | Get the force field parameters for a torsion-torsion term. |
    | `setTorsionTorsionParameters` | Set the force field parameters for a torsion-torsion term. |
    | `getTorsionTorsionGrid` | Get the torsion-torsion grid at the specified index |
    | `setTorsionTorsionGrid` | Set the torsion-torsion grid at the specified index |
    | `usesPeriodicBoundaryConditions` | Returns whether or not this force makes use of periodic boundary conditions. |

    `AmoebaTorsionTorsionForce`(void)¶
    :   Create an `AmoebaTorsionTorsionForce`.

    int `getNumTorsionTorsions`(void) *const*¶
    :   Get the number of torsion-torsion terms in the potential function

    int `getNumTorsionTorsionGrids`(void) *const*¶
    :   Get the number of torsion-torsion grids

    int `addTorsionTorsion`(int *particle1*, int *particle2*, int *particle3*, int *particle4*, int *particle5*, int *chiralCheckAtomIndex*, int *gridIndex*)¶
    :   Add a torsion-torsion term to the force field.

        |  |  |
        | --- | --- |
        | Parameters: |  |

        - **particle1** – the index of the first particle connected by the torsion-torsion
        - **particle2** – the index of the second particle connected by the torsion-torsion
        - **particle3** – the index of the third particle connected by the torsion-torsion
        - **particle4** – the index of the fourth particle connected by the torsion-torsion
        - **particle5** – the index of the fifth particle connected by the torsion-torsion
        - **chiralCheckAtomIndex** – the index of the particle connected to particle3, but not particle2 or particle4 to be used in chirality check
        - **gridIndex** – the index to the grid to be used

        |  |  |
        | --- | --- |
        | Returns: | the index of the torsion-torsion that was added |

    void `getTorsionTorsionParameters`(int *index*, int &*particle1*, int &*particle2*, int &*particle3*, int &*particle4*, int &*particle5*, int &*chiralCheckAtomIndex*, int &*gridIndex*) *const*¶
    :   Get the force field parameters for a torsion-torsion term.

        |  |  |
        | --- | --- |
        | Parameters: |  |

        - **index** – the index of the torsion-torsion for which to get parameters
        - **particle1** – [out] the index of the first particle connected by the torsion-torsion
        - **particle2** – [out] the index of the second particle connected by the torsion-torsion
        - **particle3** – [out] the index of the third particle connected by the torsion-torsion
        - **particle4** – [out] the index of the fourth particle connected by the torsion-torsion
        - **particle5** – [out] the index of the fifth particle connected by the torsion-torsion
        - **chiralCheckAtomIndex** – [out] the index of the particle connected to particle3, but not particle2 or particle4 to be used in chirality check
        - **gridIndex** – [out] the grid index

    void `setTorsionTorsionParameters`(int *index*, int *particle1*, int *particle2*, int *particle3*, int *particle4*, int *particle5*, int *chiralCheckAtomIndex*, int *gridIndex*)¶
    :   Set the force field parameters for a torsion-torsion term.

        |  |  |
        | --- | --- |
        | Parameters: |  |

        - **index** – the index of the torsion-torsion for which to set parameters
        - **particle1** – the index of the first particle connected by the torsion-torsion
        - **particle2** – the index of the second particle connected by the torsion-torsion
        - **particle3** – the index of the third particle connected by the torsion-torsion
        - **particle4** – the index of the fourth particle connected by the torsion-torsion
        - **particle5** – the index of the fifth particle connected by the torsion-torsion
        - **chiralCheckAtomIndex** – the index of the particle connected to particle3, but not particle2 or particle4 to be used in chirality check
        - **gridIndex** – the grid index

    *const* std::vector<std::vector<std::vector<double>>> &`getTorsionTorsionGrid`(int *index*) *const*¶
    :   Get the torsion-torsion grid at the specified index

        |  |  |
        | --- | --- |
        | Parameters: |  |

        - **index** – the grid index

        |  |  |
        | --- | --- |
        | Returns: | grid return grid reference |

    void `setTorsionTorsionGrid`(int *index*, *const* std::vector<std::vector<std::vector<double>>> &*grid*)¶
    :   Set the torsion-torsion grid at the specified index

        |  |  |
        | --- | --- |
        | Parameters: |  |

        - **index** – the index of the torsion-torsion for which to get parameters
        - **grid** – either 3 or 6 values may be specified per grid point. If the derivatives are omitted, they are calculated automatically by fitting a 2D spline to the energies. grid[x][y][0] = x value grid[x][y][1] = y value grid[x][y][2] = energy grid[x][y][3] = dEdx value grid[x][y][4] = dEdy value grid[x][y][5] = dEd(xy) value

    bool `usesPeriodicBoundaryConditions`() *const*¶
    :   Returns whether or not this force makes use of periodic boundary conditions.

        |  |  |
        | --- | --- |
        | Returns: | true if nonbondedMethod uses PBC and false otherwise |

High performance molecular simulation on GPUs


### Navigation

- Core Objects
- Forces
- Integrators
- Extras

©2015, Stanford University and the Authors.
|
Powered by Sphinx 1.3.1
& Alabaster 0.7.7
|
Page source
